# A collaborative chain out of phase

**DOI:** 10.5334/ijic.858

**Published:** 2013-03-14

**Authors:** Bård Paulsen, Tor Inge Romøren, Anders Grimsmo

**Affiliations:** SINTEF Technology and Society, Department of Health Research, P.O. Box 4760, 7465 Trondheim, Norway; Centre for Care Research, Gjøvik University College, P.O. Box 191, 2802 Gjøvik, Norway; Faculty of Medicine, Department of Public Health and General Practice, Norwegian University of Science and Technology, P.O. Box 8905, MTFS, 7491 Trondheim, Norway

**Keywords:** collaboration, integrated care, patient discharge, organizational culture, cross organizational communication

## Abstract

**Introduction:**

The aim of this study is to explore the obstacles to collaborations between nurses in hospital and municipal care in the discharge of hospital patients who need continuing care.

**Methods:**

First, we conducted in-depth interviews of nurses in hospitals and nurses in municipal care. Second, we developed questionnaires and distributed them to a representative sample of Norwegian municipalities to study the representativeness of the most important findings from the interviews.

**Results:**

Municipal care nurses reported that the information they receive from hospital departments usually is insufficient for a complete understanding of a patient’s needs. Formal discharge reports from hospital serve as a post factum formalization and authorization of information collected by municipal nurses in an ad hoc fashion and via oral communication. Typically, formal information routines are out of phase with the information needed by municipal care professionals.

**Conclusions:**

Hospital information provided at discharge is neither sufficient nor timely with respect to the information needs of nurses in municipal care. Organizational efforts and the use of information technology might ease some obstacles, but several problems will remain because of differences in professional orientation and the contexts of care delivery.

## Introduction

The process of a patient discharge from hospital starts when doctors decide that hospital-based medical treatment is no longer necessary for a patient. If the patient still needs professional care, hospital and municipal care nurses collaborate to ensure that the transfer to municipal-based care is properly planned. A timely exchange of sufficient information between the personnel involved is a prerequisite for co-ordination of the discharge process and for the planning of care outside of the hospital [1, 2]. Good planning and the co-ordination of discharge processes for frail patients are associated with higher patient satisfaction, fewer problems experienced by patients, and fewer unscheduled returns to hospitals [3, 4]. This paper explores the effects of organizational and cultural obstacles to collaboration between hospital and municipal care nurses in the discharge of frail and elderly patients from hospital to local care in a Norwegian setting.

Health care in Norway is divided into two distinct levels politically, administratively and financially. Organizations that provide specialist health care services (in hospitals or ambulatory) are owned and financed by the Ministry of Health and Care Services and managed by four regional health authorities. In contrast, primary health care is organized and financed by the municipalities. Health care expenditures at the two levels are roughly equal. The municipal responsibility comprises general practitioner (GP) services, out-of-hour services, maternal and child health centres, home care services and nursing homes [5]. All residents in Norway are entitled to a GP, and the GPs function as single-point gatekeepers. Home care services and nursing homes offer nursing and therapeutic procedures, medical services, rehabilitation, personal care, support services and terminal care.

Health service is heavily dependent on several well-functioning collaborative chains, one of the more important of which is the discharge of patients from hospital care to municipal care. Terms such as ‘episode of care’, ‘clinical pathways’, ‘patient trajectory’ and ‘integrated care’ reflect the growing interest in developing conceptual tools for patient-centred analyses of the functional links between the separate elements of the health care system [6–8]. An old (but still very useful) definition of an episode of care is given by Solon et al.: “A block of one or more medical services received by an individual during a period of relatively continuous contact with one or more providers of service, in relation to a particular medical problem or situation” [9, p. 403–404]. According to Solon, an episode of care is a process that covers a certain time span in which certain actors in the health care system are organized in a certain way and in a certain order around an individual patient. The quality of this process is measured by the continuity of care provided, which depends upon how well the actors involved collaborate. It is important, then, to understand the challenges to collaboration in this process model of episodes of care and how the actors involved overcome them.

The quality of collaboration is closely related to some key factors in the organizational situation in which collaboration takes place. Collaboration may be hampered by factors such as differences in organizational culture, conflicting professional attitudes and the lack of economic incentives [10, 11]. The timing, organization and distribution of tasks among actors involved will determine what kinds of interactions are needed and, in turn, the obstacles to interactions that may occur. Inspired by Thompson’s contributions to our understanding of the links between organizational technology and organizational behaviour [12], Ulrica Nylén distinguishes between three different collaborative structures: “(1): Relatively independent work performed in a sequential flow of tasks. (2): Reciprocal encounters between actors. (3): Close face-to-face interaction for joint intervention” [13, p. 146]. These three collaborative structures differ regarding the intensity of contacts and perceived gains by the actors involved. In a team organization, in which people meet face-to-face to perform joint actions, mutually adaptive behaviour depends upon the extent to which the actors understand each other’s roles and ways of thinking [10, 14–16]. The members of the team depend upon each other to perform their tasks, and reciprocity (of some kind) is an important motivation for participation. However, when work is organized into a sequential flow of tasks (a collaborative chain), the actors involved relate to each other asymmetrically. Each actor in the process is responsible for certain tasks, after which he or she transfers the responsibility for the patient to the next actor in the collaborative chain. Each actor’s service end-point is the starting point for the next actor in the chain. The last actor in the chain depends heavily on what the preceding actors in the chain have done, in a way that is unlike their dependence on him or her. In their study of collaborative patterns between the actors in the chain of care for expecting and new parents, Barimani and Hylander [17] concluded that the most important explanatory factor for attitudes towards collaboration was an actor’s position in the collaborative chain. Professional gain and collaborative profit were greatest for the last actor in the chain, for whom the actors in the first and middle positions were facilitators and information providers. As collaborative gains are asymmetrically divided, so too will be the motivation for collaborative efforts in the chain. If we assume that the discharge of elderly patients (who need care) from hospitals to community care is an asymmetrical process, then we may also assume that the gains of collaboration will be most important for nurses in municipal care. In preparing to care for a new patient, they depend upon relevant information about the patient’s needs. If the motivation for collaboration is asymmetrical across actors, formalized procedures may be established [13]. However, formalized procedures are not always sufficient to fill the information gap. The process of discharging an elderly patient from hospital to municipal care involves a journey across the boundaries of very different organizations and economic structures. In the information exchange between hospital nurses and home care nurses when a patient is discharged, there may be very different assessments of the content of the information exchanged [18, 19]. Hellesø states that the “timely transfer of contemporaneous and relevant information across the organizations is important to ensure effective patient care. However, providers in different organizational structures have different perspectives, work in different situations and have varying experience” [18, p. 2]. An organizational culture, characterized by shared values, ideologies and styles of work, influences the way its members think and act [20–23]. Differences in organizational culture, then, may create barriers to effective communication between different actors in a collaborative chain that bridges organizations.

Based on our theoretical considerations, we assume that there are several inherent obstacles to seamless patient trajectories and the continuity of care when patients are discharged from hospitals to municipal care. Interactions between hospital nurses and municipal care nurses have the characteristics of a collaborative chain, and the collaborative gains and collaborative motivations of the actors involved tend to be asymmetrical. The nurses are parts of different organizations with different types of internal structures. In a two-level health care system such as Norway has, nurses in hospitals and nurses in municipal care are subject to different regulations and reimbursement systems. Their professional staffing is different, as is their relationship to their patients. Despite their common professional identities, we can assume that hospital nurses and municipal care nurses pursue different professional goals in accordance with their different organizational cultures, as several authors have mentioned previously [20–23]. Considering the potential deficiencies in collaborative health care chains, there are important questions regarding how the actors involved overcome these deficiencies to perform their specific tasks and fulfil their professional obligations to the patients whom they serve. In our study of discharge procedures, we focused on the following research questions:

- Who initiates information exchanges during the hospital discharge process, and how do they do it?
- To what extent are formalized procedures for information exchange in patient discharges considered relevant, sufficient and timely for planning patient care in municipal care settings?
- What is the interplay between formalized procedures and informal contacts between nurses in hospitals and municipal care during the discharge process?

## Materials and methods

Our study is based on qualitative interviews and a postal survey. First, we conducted a series of interviews with strategically sampled key informants. The sample included nursing personnel in institutional and home-based long-term care in three municipalities, and nurses in the three hospitals that serve these municipalities. We developed a postal survey based on the findings from the qualitative interviews, to study support for the main findings among nursing personnel in Norwegian municipal care.

The sites we selected for qualitative analysis represent three different types of municipalities. One important criterion was different geographical relationships to their hospitals, as we consider geographical distance to be an important factor in health care collaboration. The first municipality, a town with 26,000 inhabitants, has a hospital. The second is a town with 21,000 inhabitants, who must travel about an hour to reach the nearest hospital. The third one is a small and remote municipality with 5000 residents, having at least a three-hour journey to the hospital. In each municipality, it was important to include nurses who were responsible for making decisions about the kind of municipal care frail patients received. We identified three important roles in the municipal system: the municipal patient co-ordinator, the manager of the largest nursing home in town, and the manager of the largest unit for home care service. In total, we interviewed nine nurses in municipal care. The patient co-ordinator (usually a nurse) receives applications for municipal care, assesses patients’ needs, and decides what level of care should be provided. When a decision is made, either a home-based health care manager or a manager in a local nursing home, according to where the patient is supposed to go, does further collaboration with the hospital concerning discharge procedures. (These three municipal roles are referred to as municipal nurses below unless otherwise specified.) We interviewed two experienced nurses in each of the three hospitals that serve these municipalities, one from a medical ward and one from an orthopaedic ward. We chose these specialties because they generally have many frail elderly patients.

We designed separate interview guides for representatives of the different occupations we selected, and we structured the interviews according to the stepwise character of the discharge process. We focused on the information needs of actors, the information exchanged between them, and we mapped how and when the various actors in the discharge process made requests for information. Additional topics introduced by the informants during interviews were followed up whenever they were relevant. The interviews were recorded and transcribed by a secretary, and the first author analysed the data based on the principles of grounded theory [24]. The goal of the analysis was to develop concepts for understanding the interplay between the actors in the discharge process and to create a set of hypotheses that could be explored in the postal survey.

Based on the hypotheses generated during the authors’ discussions of the results from the qualitative analysis, we designed a set of questionnaires and distributed them to key municipal care workers in a representative sample of Norwegian municipalities. Prior to distribution, we pre-tested the questionnaires on a sample of informants from the qualitative phase. The paper-based survey was distributed via postal service to a sample of 110 municipalities (26% of all municipalities in Norway). Our sample corresponded fairly well with the overall municipal structure in Norway with respect to the size and geographical distribution of municipalities. In each municipality, we distributed the questionnaires to the same key personnel identified in the qualitative part of our analysis: the local patient co-ordinator, a manager of a local nursing home and a manager of home care services. A reminder was sent by post. Of the 110 municipalities we sent questionnaires to, we received 47 answers from patient co-ordinators (response rate: 42%), 55 from managers of nursing homes (response rate 50%) and 53 from managers of home-based care (response rate: 48%). The lower response rate for patient co-ordinators was expected because of variations in municipal organizations. Not all municipalities have a patient co-ordinator.

## Results

### First warning

The patient discharge process usually starts with a notification from the hospital to a key representative of local care in the municipality where the patient lives, typically a patient co-ordinator (Table 1). Of our municipal care respondents, 25% said they usually received the initial notification during the patient’s first or second day in the hospital, whereas 51% reported that this notification usually arrived when discharge was close. In addition, 10% answered ‘other’ and 15% answered, ‘Don’t know’. The qualitative interviews revealed contradictory views regarding early warning procedures. A hospital nurse said that her ward usually sent an ‘early warning’ to the patient’s municipality town during the patient’s first day in the hospital:

“We write a notification, and at the same time we make a phone call to the local care and tell them that we have a patient from their municipality, that we have sent you a notification, and you will have more information within the next few days… Then we speed things up a little to make the town take some action…” (Hospital nurse)

An early note, usually by post, is supposed to give the municipalities necessary lead-time for planning and preparation. On the other hand, the recipient in municipal care very often considers the information given at this early phase of the patient’s hospital stay premature, and not very useful:

“We receive a notification from the hospital with some information about the patient… This notification, however, contains very little information about the patient. It is all too early.” (Municipal nurse)

This statement was supported by the survey data. Only 23% of the long-term municipal care respondents agreed that the information in such a notification was sufficient to decide what the patient’s health care needs would be after discharge from the hospital.

### Municipal nurses trying to complete patient information

The early warning usually prompts municipal nurses to collect more information. In particular, municipal nurses who work in patients’ homes need as much information as possible about what the patient’s functional, medical and mental status will be on arrival:

“I need a complete picture. To what degree can the patient help himself? Is he able to care for himself? Does he need help to eat? Does he sleep at night? Is he anxious? Does he need attendance at night?” (Municipal nurse)

Very often, municipal nurses consider the information they get from the hospital to be insufficient and they must make their own inquiries. The interplay between nurses on both sides of the exchange may be a process of stepwise consideration of the available information:

“The diagnosis may cause some thought for the person (in the town) receiving our message.” (Hospital nurse)

A municipal nurse confirmed that the diagnosis was a starting point for her own inquiries. She described the information-seeking process she uses to collect some basic information, ask new questions, and interpret information relative to her own professional knowledge of home care services. This indicates that she often calls the hospital for additional information. The municipal nurse must take initiative, with the hospital nurse playing a more passive role in merely answering questions. Collecting information may be a stepwise process, including a series of phone calls to ask questions, time to consider the implications of the answers, and possibly additional inquiries, all in an effort to get a complete picture of the patient’s needs. The municipal nurse describes this as a necessary process through which she tries to relate the information acquired from observations of the patient in the hospital setting to the very different situation of taking care of a frail, elderly patient in his or her own home:

“Of course, they answer all my questions. And, you know, having been so long in this business, I have become so experienced that I understand that, in this case, a short stay in a nursing home is necessary.” (Municipal nurse)

### Collaboration across organizational borders: finding the right informant

Table 2 reports on data from the questionnaire survey. According to the experiences of municipal nurses, a liaison nurse (or any other appointed contact person) is uncommon in hospitals. Municipal nurses describe situations in which they have to contact several people in the hospital to get information about a patient. According to municipal nurses, finding the right person may be difficult. On the other hand, they do not attribute the problems they have finding the right person to the unwillingness or bad attitudes of hospital nurses. On the contrary, a majority of our respondents report that they are always met with kindness and goodwill.

A hospital nurse explained the consequences of internal hospital organization on the patient–nurse relationship and information handling for individual patients:

“This will vary a lot on our side, because there are several nurses responsible for this. The responsibility for doctor’s rounds and patient discharges rotates between the nurses on a weekly basis, so many nurses will be involved. None of us does these things in the same way, I think.” (Hospital nurse)

A nurse working in another hospital gave the following description:

“We are organized in different teams, one for each of the corridors. We have a team leader taking care of medicines and co-ordination and organizing the teams. And so, we are responsible for one, two or three rooms, according to how much care patients in the different rooms need.” (Hospital nurse)

From the municipal nurses’ point of view, hospital shift work makes getting information very complicated:

“There may be a lot of persons involved, right? Maybe I have talked to a nurse (in the hospital). And then, next time, she has gone home, because the patient was not discharged before the afternoon. And at that time, the nurse I talked with is not there. Then, you know, it is a problem to get information through the system” (Municipal nurse)

In addition, internal hospital procedures for the exchange of information were insufficient according to the municipal nurses. Only 27% had the impression that hospital nurses communicate well from one shift to the next (Table 2).

### The day of discharge: formal procedures for information exchange are decoupled from the tasks they are supposed to support

When a patient is discharged from hospital, a medical discharge letter and a nursing report usually accompany him or her. Both types of information are considered necessary for the continued care of the patient. In our survey, however, 74% of the nursing home and home care services managers agreed with the following statement:

“We have already received most of the information in the (hospital) nursing reports through contacts with the hospital during the patient’s stay.” (Municipal nurse)

The responses to the above statement of the 108 people who answered: ‘always’: 3%; ‘usually’: 71%; ‘sometimes’: 22%; ‘never’: 1%; and “don’t know/no opinion”: 3%.

In the interviews, a nursing home manager explained why these routine documents delivered at discharge came too late to serve information needs in her preparations for a new patient to come:

“We do that (make our phone calls beforehand) to be sure that everything is settled when the discharge summary and nursing report arrives with the patient… We have to be very careful with the collection of medical information to make sure that we have it all settled when the patient arrives. We do not have a big stock (of medicine) in the nursing home.” (Manager, nursing home)

Seamless care implies careful preparations for a patient’s arrival, either at home or at a nursing home. When a patient is being discharged, a necessary supply of medication must be available and housing facilities and family support must be considered as well. When judged with respect to the information municipal nurses need for planning and preparation, the hospital information that follows patients at discharge is out of phase. Effectively, it is a post factum formalization and authorization of information that has already been collected in a more informal, ad hoc fashion, usually via oral communication between the municipal nurses and a changing series of partners on the hospital ward. However, it may have a quality assurance purpose and serve as a verification of patient information already collected:

“Yes, those are the things I already have made phone calls and asked about—and which I already know. But it is important that we get the one from the doctor, you know, because he writes what medication the patient shall have, and that stuff…” (Municipal nurse)

### Organizational borders, professional attitudes and professional mandates

When a patient leaves the hospital, the responsibility for his or her care shifts from the hospital nurses to the municipal care nurses. It is municipal care personnel who must determine the type and extent of services the patient needs, within the legal, organizational and economic framework of the local municipality. In principle, the boundaries and reciprocal limitations of hospital and municipal nursing responsibilities are straightforward. In practice, however, this hand over of responsibilities from one group to the other may be difficult and occasionally cause conflicts (Table 3).

About half of the municipal care nurses in our sample felt that hospital nurses regularly tried to overrule them. In the interviews, many of our informants in municipal care described difficult situations that can occur at discharge. The patient or the patient’s family may confront them with ‘promises’ made by hospital nurses about the types of service they should receive when they arrive at their municipal care destination. Sometimes patients are told that they are ‘entitled’ to certain municipal care services. Some of our hospital informants acknowledged this too:

“We have been told by the municipal nurses that they thought that in earlier days we were too eager to try to decide that the patient needed this or that (after discharge), but now the municipal nurses have said they want to decide for themselves.” (Hospital nurse)

“Sometimes patients’ relatives use us to put pressure on the municipal care services. They realize that this (service application) will not go through locally, ‘Will you please help us so that he gets it?’” (Hospital nurse)

## Discussion

Our study of the discharge process for hospital patients who need continued care has revealed an asymmetric pattern of collaboration in which discharge information from hospital is out of phase with the municipal care tasks it is supposed to support. The discharge process is triggered by a notification from the hospital that a patient is being discharged. Municipal care nurses describe this notification as information-poor. Very often, it is sent during the last days of a patient’s hospital stay. An early warning sent during the patient’s first days gives nurses in municipal care more time to collect additional information and for planning, but it is a weaker basis for planning, because hospital nurses need some time to get to know the patient. Once they receive a warning from the hospital, municipal nurses often start collecting the information they need to get a complete picture of the patient’s needs so they can choose an appropriate level of service and prepare for the patient’s discharge. In this collaborative process, municipal care nurses describe themselves as the active actors, and they see hospital nurses as passive actors simply answering questions. Hospital scheduling rarely gives municipal care nurses a single collaborative partner to contact for information on a given patient, and sometimes it is not clear who has the information they need.

It is not until the day of discharge that the information needed by municipal care personnel for planning and practical preparations is carefully organized, prepared and sent along with the patient. By then, however, municipal nurses typically have already obtained the same information informally, and the formal information exchange procedures merely serve to legitimize it. The formal procedures for the exchange of information are decoupled from the actual information needs, and this forces municipal care nurses to rely on less formal means of acquisition that are less secure, outside of quality control and more time-consuming to complete. The formalized procedures for information collection and organization are rooted in hospital routines and reporting systems, and are not based on the information needs of the hospital’s collaborative partners in municipal health care. This point is underlined by the municipal care nurse’s statement that contact partners in hospitals appear to be shifting, unreliable and random. To date, the organization and professional culture of hospitals has not encouraged hospital nurses to take a more active role in their collaborations with municipal care nurses.

According to the theory of collaborative gains, which indicates that gains are concentrated in the last actor in the collaborative chain [17], the asymmetry of the collaborative health care chain may be an important explanation for the patterns observed. For nurses working in municipal care, relevant and sufficient information is crucial to their seamless provision of care for discharged patients. Their gain depends upon hospital nurses collecting information for them. For hospital nurses, on the other hand, no information exchange or mutual action with municipal care nurses is necessary for their work. Their goal is the prompt discharge of a patient who no longer requires their services. However, we need more research on the effects of organisational conditions within the hospital ward on their role as informants and collaborators for nurses outside hospital. Hospital wards organize their nursing staff in different ways [25, 26]. Differences in ward organization may have important consequences for nurses’ access to relevant and updated information on patients. The interplay between nurses and doctors, who are an important source for patient information, may be another important factor [27]. A better understanding of the communicative culture within a hospital may be important to our understanding of hospital nurses’ role in cross-organizational collaboration. Coiera and Thombs, making a study of communication patterns in hospital, concluded that nurses and doctors preferred oral communication, instead of written information on paper or electronically. This made the hospital ward a very interruptive workplace [28]. However, a communicative culture based on ad hoc oral communication makes the one who needs information the active part in the collaborative process, similar to what we observed in the cross organizational communication between municipal care nurses and nurses in hospital.

The collaborative chain responsible for the discharge of patients from hospitals to municipal care seems to have occurred naturally, characterized by processes in which municipal nurses try to solve their information needs through incremental or adaptive muddling through in their collaborations with hospital nurses [29–31]. Therefore, up to now, diversity has been one of the most thorough and persistent characteristics of hospital discharge procedures. The cross organisational collaboration between nurses at discharge has received very little attention. This is very different from the doctor-doctor communication associated with hospitalisation, which in Norway and in most countries are much better regulated, and have been subject to more research internationally.

Attempts have been made to structure the collaboration between hospital and municipal care nurses by nominating dedicated persons on either or both sides to manage the process. Attempts to develop the role of a hospital/municipal liaison nurse started as early as the 1960s [32]. In most such cases, hospitals have hired professionals from municipal care to be liaison nurses, but an evaluation of the effects of these efforts across wards and hospitals is lacking [33]. Another initiative has been to organize different forms of multidisciplinary teams to assist at discharge and follow-up. Such organizations, however, often are fragile and volatile [34]. Some positive results have been seen in trials where personnel from specialist care take part establishing home-based municipal care immediately after discharge [35, 36]. However, many of these trials have focused on patients with a single diagnosis. This approach has its limitations when most long-term patients have multiple diagnoses. There is also the risk of fragmenting primary care when it is organized as an extension of the multiple specialties in hospitals. Care co-operation models with a broader scope are emerging [37], and some interesting initiatives are rooted in primary care [38].

One important characteristic of the collaborative chain in health care is that it is information-driven [39]. In contrast to manufacturing production lines, little happens unless decisions and information are communicated to all actors first. The interviews in this study have demonstrated the problems that arise when the flow of information is out of phase in the hospital discharge process. However, rapidly developing information technologies present new possibilities, as well as a technology-driven motivation to analyse and rearrange traditional collaborative chains. The most novel possibility is the sharing of information such as electronic record systems that give municipal nurses direct access to their patients’ health records so they can follow their progress. Thus far, however, the *electronic sharing of records* across health care organizations has not enhanced collaboration [40–42]. Systems for *exchanging information* are probably more effective in health care co-ordination [28]. One reason is that sharing electronic health records across health care organizations only makes data and information about patients more easily available, whereas the exchange of information in summary reports such as referrals and discharge letters also includes the provider’s *knowledge* about the patient. This is efficient and sometimes crucial for correct decisions.

From the description given by the nurses about the collaboration at discharge, information-seeking behaviour was disruptive and time-consuming for both parties. The problems with timing, order and shifting contacts suggest the introduction of asynchronous email-like electronic communication. A liaison nurse or another nominated person could answer the requests coming from municipal care nurses at an appropriate time for both parties.

We are not sure if email-like communication will be good enough. It is difficult to compensate for the richness, flexibility, and immediacy of direct conversations, as information can be corrected and supplemented in real time. Although the stepwise collection of information described by the municipal nurses may not be optimal, they are in charge of such information exchanges. Their control of the types and relevance of the information they receive could be reduced by using only written (or electronic) communication, as their experience with the formal nursing reports and discharge letters indicates. We believe this control is important for their follow-up success with patients because, as we saw, hospital nurses and municipal nurses have different views about what is important and necessary information in the transition of patients from hospital to municipal care. The interviews and the literature indicate that these different views reflect differences in their professional orientations and the contexts in which care is delivered. In addition, these different views also reflect differences in the patient role in the hospital and at home. A second problem is that sometimes the fluid understanding of the reciprocal limitations of their respective mandates is a source of irritation and discontent for the personnel involved.

## Strengths and limitations

This study is based on a series of qualitative interviews and a postal questionnaire derived from the interviews. The qualitative interviews allowed us to detect important aspects of the collaborative process, and the survey allowed us to increase the generalizability of our findings. However, the low response rate (from 42% to 50% for the three groups studied) must be considered. The survey indicates that the findings from the in-depth interviews are widespread and representative of experiences in the transition of patients from hospital to municipal care. However, there are tremendous differences in the organization of health care. Generalizing the findings from this survey to other countries and organizations should be done with caution. On the other hand, our impression is that the segregation between primary care and specialist care is especially clear in Norway. This probably makes the cultural and professional differences that we found more visible than in other settings, even if they are the same.

## Conclusion

To realize the concept of integrated care, health service depends upon effective collaborative chains across organizational borders. Our study demonstrates the organizational challenges in the creation of collaborative chains. They are hampered by incongruent or insufficient internal procedures, differing organizational cultures and unclear mandates between the actors involved. More research is needed on the effects of ward organization on cross-organizational collaborative patterns.

It is important for health care service providers to find effective means to overcome these problems. The introduction of information technology will help to eliminate some of the known obstacles to the co-ordination of care at discharge. However, we suspect that information technology will only partly replace oral communication, and it will not make the formal documents at discharge superfluous. It will become a third and supplementary means of exchanging patient information. It is important to discuss how information technology could better integrate these means of communication. When introducing information technology, it is crucial to consider the organizational aspects of the information exchanged and the collaborations involved.

## Reviewers

**Johan Berlin**, PhD, Associate Professor, Senior Lecturer, Department of Social and Behavioural Studies, University West, SE 461 86 Trollhättan, Sweden

**Mark Govers**, Faculty of Health, Medicine and Life Sciences, Department of HOPE, Maastricht University, School for Public Health and Primary Care (CAPHRI), P.O. Box 616, 6200 MD Maastricht, Netherlands

**Line Melby,** Researcher, Institute for Health and Society, University of Oslo, Box 1130, Blindern, 0318 Oslo, Norway

## Figures and Tables

**Table 1.**
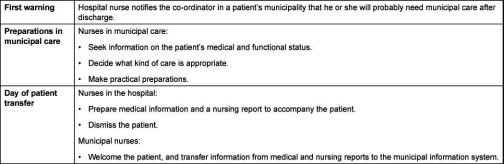
Information exchange in the process of discharging patients from hospital care to municipal care.

**Table 2.**
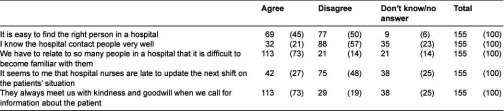
Finding a contact person in a hospital. Number of municipal nurses and (%).

**Table 3.**
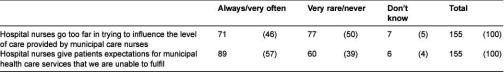
Do hospital nurses try to influence municipal care decisions? Number of municipal nurses and (%).
